# Evaluation of the clinical efficacy of potassium nitrate desensitizing mouthwash and a toothpaste in the treatment of dentinal hypersensitivity

**DOI:** 10.4317/jced.50665

**Published:** 2012-02-01

**Authors:** Sunita Sharma, Neetha J. Shetty, Ashita Uppoor

**Affiliations:** 1 Specialist Resident, Department of Periodontology. Manipal College of Dental Sciences, Mangalore; 2 Associate Professor, Department of Periodontology. Manipal College of Dental Sciences, Mangalore; 3 Professor and Head, Department of Periodontology. Manipal College of Dental Sciences, Mangalore

## Abstract

Potassium Nitrate has been used as a desensitizing agent to treat dentinal hypersensitivity. The effectiveness of a potassium nitrate is evaluated both in the form of a toothpaste and a mouthwash in a clinical study. Thirty patients were assessed using evaporative stimuli and thermal stimuli and response was evaluated using Visual Analogue Scale at baseline, at 2 weeks and 4 weeks. The patients were divided into. group I: fifteen patients who used toothpaste containing 5% potassium nitrate, sodium fluoride, xylitol and triclosan, group II: Fifteen patients who used mouthwash containing 3% potassium nitrate, sodium fluoride, xylitol and triclosan . The results of both the assessment methods indicated that potassium nitrate toothpaste as well as mouthwash showed statistically significant decrease in the sensitivity score on a Visual Analogue Scale. This was effective in reducing the symptoms of dentinal hypersensitivity when used either as toothpaste or as a mouthwash. But, there were no statistically significant differences between the groups, although both were effective in the treatment of hypersensitivity.

** Key words:**Dentinal hypersensitivity, potassium nitrate toothpaste, potassium nitrate mouthwash, desensitizing agents.

## Introduction

Dentinal Hypersensitivity (DH) is characterized by short sharp pain arising from exposed dentin in response to stimuli typically thermal, evaporative, tactile, osmotic or chemical-that cannot be ascribed to any other dental defect or disease. (1) DH usually is diagnosed after other possible conditions have been eliminated. Chipped or fractured teeth, cracked cusps, carious lesions, leaky restorations and palatogingival grooves are alternative causes of pain. Currently, explaining dentinal hypersensitivity favors the hydrodynamic theory originally postulated in the nineteenth century and developed later by Brannstrom in 1963. (2,3) This theory states that dentine hypersensitivity may be caused by movement of the dentinal tubule contents. An increased outward fluid flow causes a pressure change across the dentine, distorting the A-δ fibres by a mechanoreceptor action, causing sharp, shooting pain.

The prevalence of DH varies from 45 to 57 percent. (2) These variations are likely due to differences in the populations studied and the methods of investigation (for example, questionnaires or clinical examinations). While DH mostly occurs in patients who are between 30 and 40 years old, it may affect patients of any age. It affects women more often than men, though the sex difference rarely is statistically significant. The condition may affect any tooth, but it most often affects canines and premolars. (2,3) Moreover, the prevalence of this condition is likely to increase in the future as individuals retain their dentitions for a longer period of time.

Since dentin has close structural and functional relationship with the dental pulp, sensitivity owing to its close proximity is natural. (4) This inherent sensitivity usually is not a problem because other tissues cover the dentin. Once there is a tissue loss such as enamel wear, loss of cementum and gingival tissue, it manifests as dentinal hypersensitivity. Microscopic examination reveals that patent dentinal tubules are more numerous and wider in hypersensitive dentin than in no sensitive dentin. (5,6)

An understanding of the hydrodynamic mechanism of dentin sensitivity provides a basis for developing desensitizing therapies. Classifying treatments for DH can be challenging because its modes of action often are unknown. It can be simpler to classify treatments according to their mode of delivery. Treatments can be self administered by the patient at home or be applied by a dental professional in the dental office. At home methods tend to be simple and inexpensive and can simultaneously treat generalized DH affecting many teeth. (7) The disadvantages of these treatments include compliance, difficulty of delivery to specific sites, slow onset of action, and requirement of continuous use. In-office treatments are more complex and generally target DH localized to one or a few teeth.

Most hypersensitive teeth are accompanied by gingival recession presumably resulting from periodontal disease, periodontal therapy, or improper brushing habits. Attempts to reduce dentine hypersensitivity have been aimed at either reducing the excitability of the nerve fibers within the pulp or occluding the open dentinal tubules. Various agents have been used as desensitizers for hypersensitive teeth including silver nitrate, fluoride, formaldehyde, strontium chloride and potassium nitrate. (8,9,10) Dentifrices containing potassium ions have been shown by several clinical studies to be effective in reducing dentine hypersensitivity and the American Dental Association Council on Dental Therapeutics has granted a Seal of Acceptance to dentifrices containing 5% potassium nitrate (Council on Dental Therapeutics 1986). Potassium ions are thought to act by blocking the action potential generated in intradental nerves. (11,12)

Potassium nitrate is used either as a toothpaste as or as a mouthwash. And, there is always a dilemma regarding whether it is effective when delivered as toothpaste or as a mouthwash. There has been evidence in literature which shows that both the formulations have therapeutic potential to alleviate dentinal hypersensitivity. But, the studies which compare the effectiveness of toothpaste and a mouthwash are rare. The present study is designed to compare the effectiveness of desensitizing toothpaste and a mouthwash , both containing potassium nitrate for the treatment of dentinal hypersensitivity .The effectiveness of any desensitizing agent also depends on patient compliance. As the toothpaste and mouthwash are two different modes of delivery we also can appreciate to which mode of delivery patient are more compliant.

## Material and Methods

This was a clinical trial conducted in Department of Periodontics. Thirty patients were selected with sensitive teeth and randomly divided into two groups .The desensitizing agents to be studied were grouped as: Group I – Patients who used toothpaste containing 5% potassium nitrate , sodium fluoride ,xylitol and triclosan(toothpaste group n=15). Group II- Patients who used mouthwash containing 3% potassium nitrate , sodium fluoride ,xylitol and triclosan (mouthwash group n=15). Patients reporting sensitivity from hot, cold, sweet, or sour or during brushing were included as subjects having dentinal hypersensitivity. Patients were excluded from the study if they had any of the following conditions.

● Subjects with history of treatment for dentin hypersensitivity

● Poor periodontal condition

● Systemic debilitating disease

● Caries or restoration in the area of hypersensitivity

● Allergy to the agents used in the study

● Patients with orthodontic appliance, crowns , bridges in the area of sensitivity

All patients were provided detailed information, both verbally and in written form, of the principles of treatment and purpose of the study. All the patients received and signed the appropriate informed consent forms. The study protocol and consent form were approved by the Institutional Ethics Committee. A single examiner was involved in examining the subjects and assessing sensitivity. All patients were given oral hygiene instructions. Patients were advised to use a new soft brush for brushing. Patients were randomly divided into group I and group II. Patients under group I were given self-applied toothpaste to be used twice daily and were instructed to brush with allocated toothpaste for 2-3 minutes. The tooth paste was to be used only in the areas which were sensitive. Patients under group II were given a self applied mouthwash to be used twice daily and were instructed to brush with non fluoridated toothpaste for 2-3 minutes twice daily, followed by rinsing with 1ml of water for 1 minute and then using 10ml of the allocated mouthwash for 1 min before spitting out. All patients were asked to return the mouth wash bottles and toothpastes at 2 and 4 weeks, at which time replacement products were provided. All patients were recalled after 2 weeks and 4 weeks for follow up and evaluated for sensitivity.

Stimuli to assess sensitivity: Evaporative stimuli i.e. a 1 second application of cold air from a dental unit syringe (at 20º±3ºC at 40 to 65 psi) was applied 1 cm away from the affected teeth to determine the participant’s baseline response and followed by thermal stimuli i.e. application of cold water maintained at a temperature of 4ºC after 5 minutes.

Evaluation of response: The participants teeth were subjectively assessed by means of a VAS(Visual Analogue Scale) .The VAS was a 10-cm line with the anchor words “no pain” (0 cm) and “intolerable pain (10 cm)” at the opposite ends. Each participant was asked to place a vertical mark on the VAS to indicate the intensity of his or her level of sensitivity after receiving stimuli.

Statistical analysis: In our study, descriptive statistics is presented as mean ±standard deviation (SD) based on the 10-cm Visual Analogue Scale (VAS). Students unpaired‘t’ test was used to compare the differences in scores between two groups. ANOVA test (Fishers’s test) calculated the mean decrease in VAS score for both groups at three point data collection time. Bonferroni multiple comparison(with repeated measures) was made to calculate the mean difference in VAS scores at different time points i.e. at baseline, at week 2 and week 4. Calculations were performed using the statistical package SPSS (Statistical Program for Social Sciences) version 11.5 SPSS Inc,chigaco)and p< 0.05 was considered to be significant.

## Results

15 subjects with dentinal hypersensitivity with the mean age of 40.40±12, out of which 7 were females and 8 were males were in group I. In group II, also there were 15 subjects out of which 5 were female and 10 males with the mean age of 40.93±11.2. The mean VAS scores for the two treatment groups after receiving air stimuli (VAS-A) and thermal stimuli (VAS-C) at baseline, at week 2 and week 4 are presented in  Table 1. At the end of 4 weeks and during the four week- evaluation period, no adverse events such as allergic reaction were reported for any of the ingredients in the desensitizing agents.

**Table 1:**
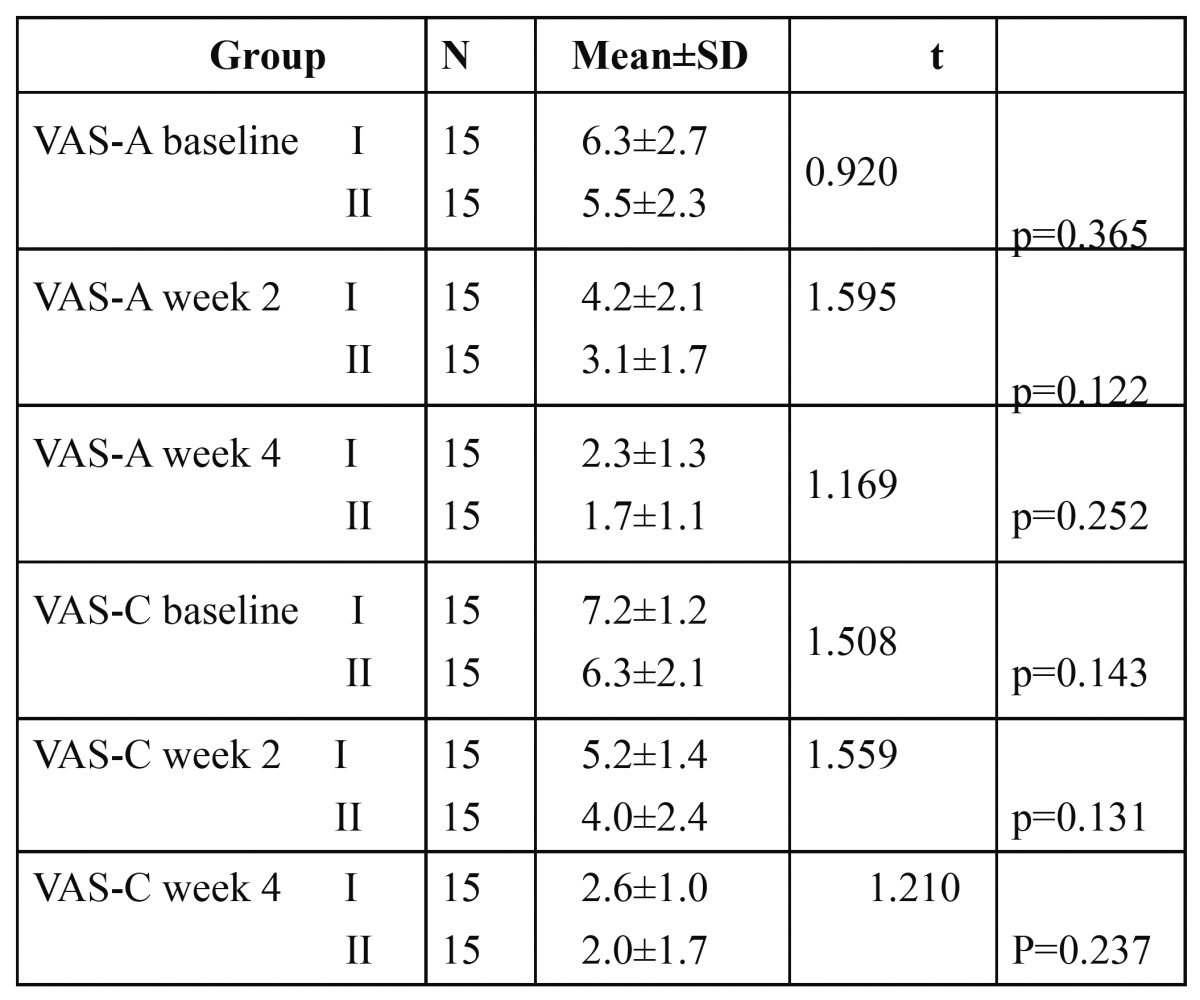
The mean VAS scores for the two treatment groups after receiving air evaporative stimuli (VAS-A) and thermal stimuli (VAS-C) at baseline , at week 2 and at week 4.

**Figure 1:**
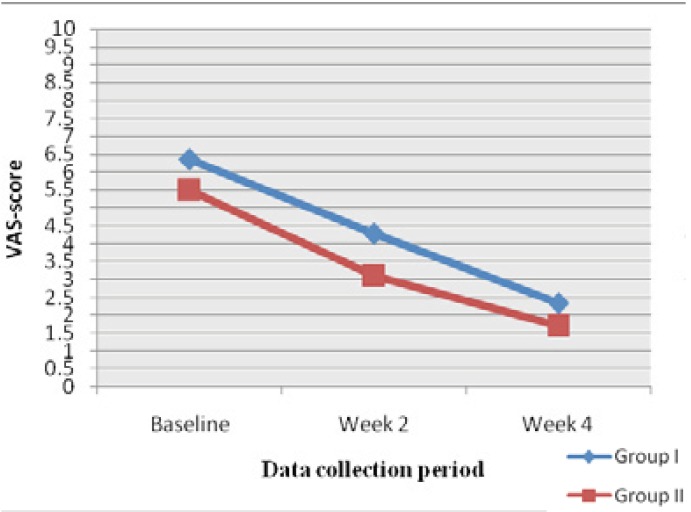
Mean VAS scores at baseline at week 2 and at week 4, in group I and group II in response to air stimuli.

**Figure 2:**
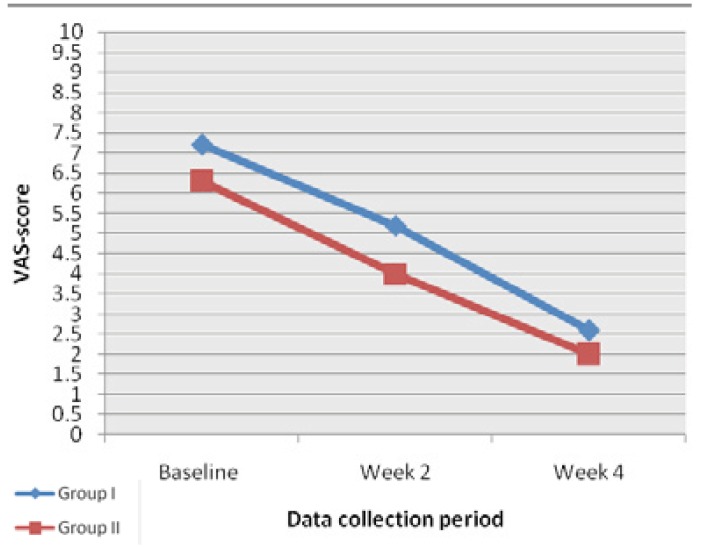
Mean VAS scores at baseline at week 2 and at week 4, in group I and group II in response to thermal stimuli.

 At baseline the mean VAS score in group I was 6.3±2.7 and 7.2±1.2 in response to air and thermal stimuli respectively. And in Group II, the mean VAS score was 5.5±2.3 and 6.3±2.1 in response to air and thermal stimuli respectively. After using the desensitizing agent, we found that all VAS scores from the post treatment periods were significantly lower in both the groups in response to both air stimuli and thermal stimuli as shown in figs. 1,2 (p<0.001). While comparing group I and group II there was no significant difference in VAS scores in all three time periods. Both tooth paste and mouthwash are effective in treating the condition.

The results showed significant decrease in VAS scores as compared to baseline in week 2 as well as in week 4 in both treatment groups with air stimuli as well as with cold water stimulation. In group I VAS score to air stimuli(VAS-A) decreased from 6.6±2.7 at baseline to 2.3±1.3 at week 4.Similarly, VAS score to cold water(VAS-C) decreased from 7.2±1.2 at baseline to 2.6±1.0 at week 4. In the patients in group II VAS-A was 5.5±2.3 at baseline which decreased to 1.7±1.1 at week 4 and VAS-C was 6.3±2.1 at baseline which also showed decreased score of 2.0±1.7 at week 4 which are depicted in  Table 2, Table 3.

**Table 2:**
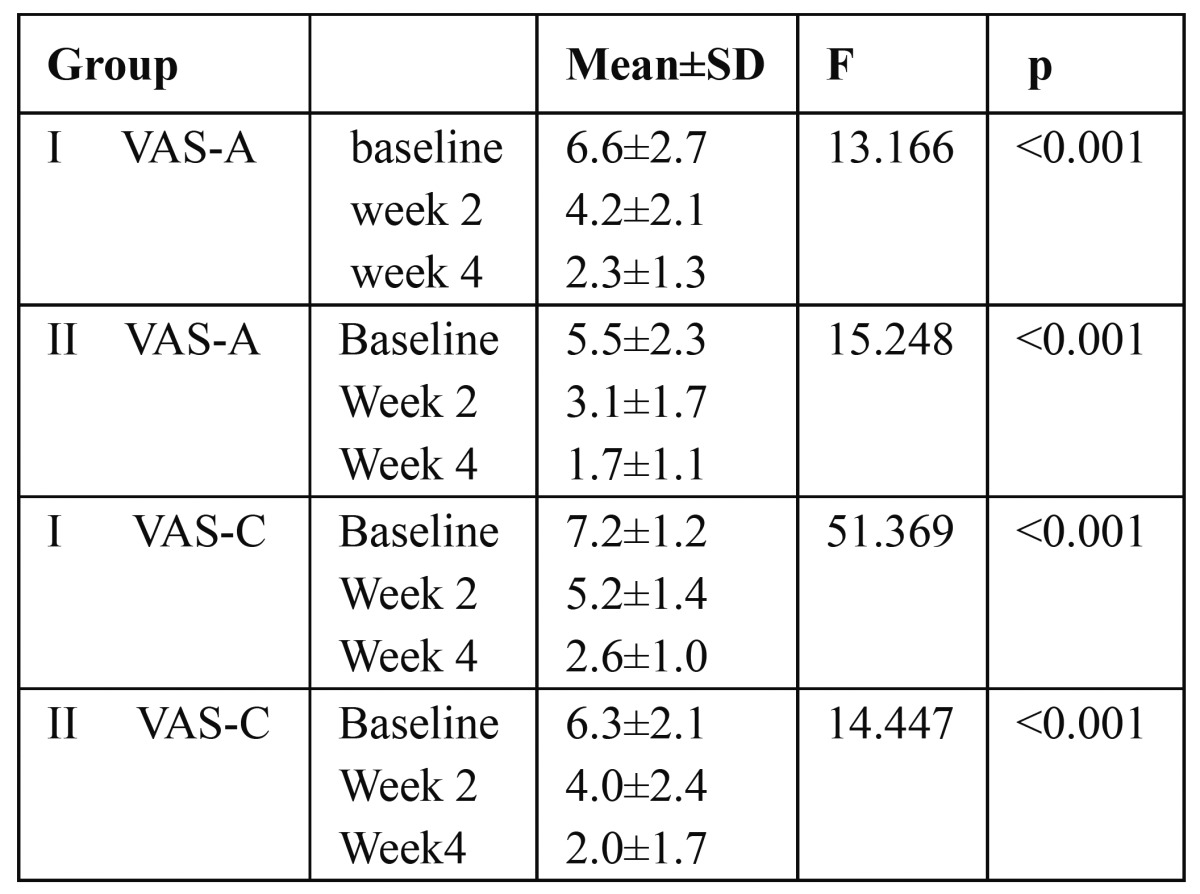
The mean VAS scores of the group using desensitizing toothpaste (group I) and group using desensitizing mouthwash (Group II) in subsequent evaluation for air stimuli and thermal stimuli compared with baseline data.

**Table 3:**
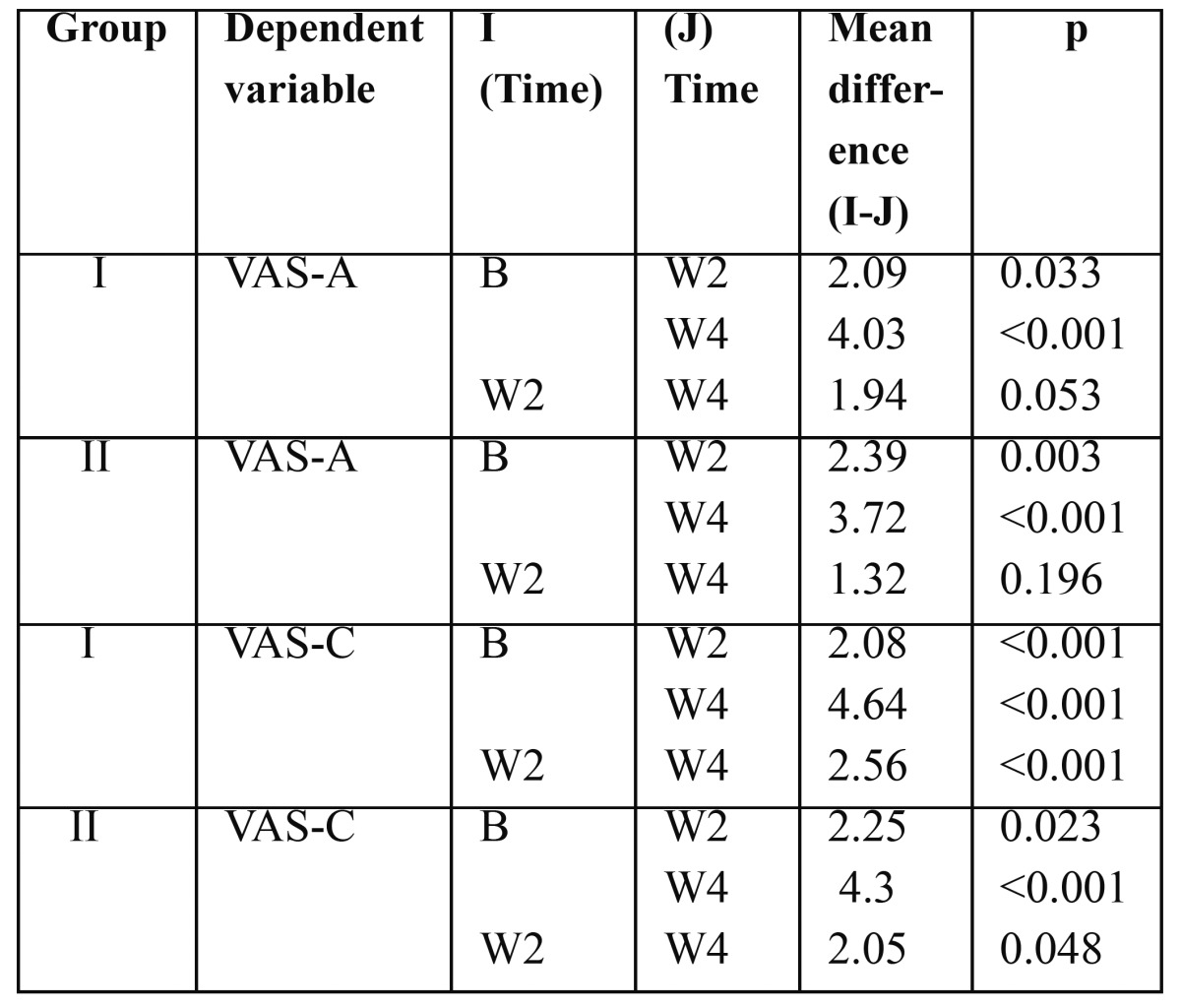
Mean difference in visual analogue scale in both groups in response to air stimuli (VAS-A) and to thermal stimuli (VAS-C) by Bonferroni multiple comparisons,at baseline (B) at week 2 (W2) and at week 4(W4).

However, the mean difference in sensitivity between week 2 and week 4 in group I and group II in response to air stimulation was 1.94 and 1.32 respectively which was statistically not significant.

## Discussion

This was a clinical study to evaluate and compare the efficacy of toothpaste containing 5% potassium nitrate, sodium fluoride, xylitol and triclosan and a mouthwash containing 3% potassium nitrate, sodium fluoride, xylitol and triclosan in the treatment of dentinal hypersensitivity.

The results of our clinical study showed that both desensitizing toothpaste and mouthwash are equally effective in reducing sensitivity within 4 weeks evaluation period, despite the different application procedure. Previous studies have reported that dentifrices containing potassium ions are effective in reducing sensitivity and the American Dental Association Council on Dental Therapeutics has granted its Seal of Acceptance to dentifrices containing 5% potassium nitrate (Council on Dental Therapeutics 1986).(13) Using ANOVA (Fischer’s test) the VAS score in response to air stimulation as well as to thermal stimulation demonstrated a significant difference in sensitivity scores over a four weeks study period. This demonstration of effectiveness of 5% potassium nitrate and 0.2% sodium fluoride toothpaste as effective desensitizing agents is in accordance with the various studies conducted in the past. (14,15) However there are very few published studies which reported the effectiveness of mouthwash containing 3%potassium nitrate and 0.2% sodium fluoride. The result of our study correlates with the findings of study by Pereira et al. 2001. (16)

The subjective nature of DH pain makes objective evaluation of it difficult. In our study, we found that both mouthwash and toothpaste were effective in reducing DH, as indicated by VAS scores .To determine the participants’ sensitivity levels in our study, we translated the subjective feedback to both air and thermal stimuli into objective data using VAS, which is the most appropriate method to use to diagnose pain levels. (17) To assess pain, we used more than one stimulus as recommended by Holland et al.1997. (18)

The use of a control group in studies investigating DH can be problematic. (18) A negative control, in which no treatment or placebo treatment is received, is an alternative; however, researchers have argued that the use of a negative control is unethical. (19) Nevertheless, most guidelines recommend that a negative control be included in clinical trials that are conducted to investigate DH. (18) Concerning the reduced DH scores in our study, an additional true placebo effect should be taken into consideration. Including a placebo group in our study might have enabled us to determine more clearly whether any of the results obtained were due to a placebo effect. Therefore, the placebo effect should be kept in mind when considering results. The abrasive components of toothpaste can also bring about tubule occlusion. (20) Since all the patients brushed with a non-fluoridated paste prior to rinsing with the allotted mouthwash, reduction in sensitivity due to brushing cannot be ruled out.

Kielbassa et al (21) evaluated the effectiveness of two fluoride agents (calcium fluoride and sodium fluoride) in reducing DH in the short term (four weeks) and long term (six and 12 months). They found that the use of these agents led to a significant reduction in DH within four weeks and resulted in constantly low sensitivity scores in the long term (six-12 months) compared with the baseline data. The reduction in sensitivity in our study demonstrates that both the mouthwash and toothpaste are effective in short term.

The therapeutic effect of any DH treatment can be attributed to many other factors, it is generally accepted that in many patients the discomfort will decrease over time without any treatment. This cessation of discomfort can be due to natural occlusion of dentin tubules, a decreased number of patent tubules, an increased incidence of reparative dentin or to seasonal changes. (22) When considering the outcomes of our study, we assume that at least some of these possible effects could have played a role in reducing dentinal hypersensitivity.

## Conclusion

Thus, the results of the present study suggest that there was an overall decrease in dentinal hypersensitivity in both groups as demonstrated by two assessment methods, over the 4-week study period. The sensitivity scores were significantly lower for the toothpaste and mouthwash using treatment groups in response to air stimuli and thermal stimuli at 2, and 4 weeks. The reduction of dentinal hypersensitivity was also clinically significant over the study period among patients who initially presented with complaint of sensitivity and later reported significant reduction of their symptoms. Therefore it can be suggested that rinsing twice daily with a 3% potassium nitrate/sodium fluoride mouthwash or brushing twice daily with a 5% potassium nitrate/sodium fluoride toothpaste may help reduce discomfort arising from dentinal hypersensitivity. But, there were no statistically significant differences between the groups, although both were effective in the treatment of hypersensitivity. However, long-term studies to facilitate better understanding of the performance of these desensitizing agents can be advocated in the future.
